# Certain Causes of Major Pelvic Troubles Traceable to Minor Gynecology

**Published:** 1890-11

**Authors:** Joseph Price

**Affiliations:** Philadelphia, Pa., Physician in Charge of the Preston Retreat; Fellow of the American Association of Obstetricians and Gynecologists, etc., etc.


Buffalo Medical and Surgical Journal

VOL. XXX. NOVEMBER, 1890. No. 4.

### Original Communications.

### CERTAIN CAUSES OF MAJOR PELVIC TROUBLES TRACEABLE TO MINOR GYNECOLOGY.[1]

BY JOSEPH PRICE, M. D., Philadelphia, Pa.,

Physician in Charge of the Preston Retreat; Fellow of the American Association of Obstetricians and Gynecologists, etc., etc.

WITH the present popular cry of "conservatism," in reference to operation in cases where it is held that all treatment should be tried previous to real surgical interference, it is worth while asking whether this preliminary treatment should not itself be abandoned in the hands of those who plead most pathetically for it. Their cry is not a scientific plea, but in most instances a *personal bid* for indulgence while they try to accomplish something, without acknowledging on the one hand that there is little or nothing to encourage them in their work, so far as results are concerned; and on the other, that there is abundant proof from the cases that have come out from under their hands, with one treatment or another, that manifold and really major surgical affections arise merely from treatment recognized as orthodox from the standpoint of minor gynecology. So far as my own experience is concerned, I do not hesitate to put minor gynecology in a casual relation with a vast amount of the necessary major pelvic surgery coming under my attention.

First among these causes may be mentioned the Emmet cervical operation. Like many other surgical operations, this, when first explained by its distinguished originator, was done in season and out, by every one, without the least consideration of its contra-indications. Very many minor tears of the cervix, in which a cosmetic effect only is obtained by operation, are made distinctly worse by operative interference. In many cases the pain becomes insufferable, from the lighting up of a dormant or unrecognized

1, Read in the Philadelphia County Medical Society, September 24, 1890.

pelvic trouble, and operation is required to undo the mischief of an unnecessary cervical closure. This fact has been recognized by Emmet himself, and he has counseled the careful selection of cases in order to escape these disastrous results. It should be set down that where there is preëxisting pelvic disease, even though slight, no cervical operation ought to be tried unless absolutely required by the condition of the patient. Another operation, which has met with much approval in many directions, and which some measure of success seems to follow in some cases, is the forcible dilatation of the cervix. It is clear that where there is antecedent inflammation of the pelvic viscera, that is of the genito-urinary system, such an operation as surgical dilatation of the cervix cannot be free from danger. In order to relieve dysmenorrhea by this procedure, it must evidently be due to stenosis of the os or cervix.

The question here arises, Can it be told, in dysmenorrhea, wherein its causes lie? Sometimes; but not infallibly. The fact is that in many women where a stenosis would be diagnosticated, there is no difficulty whatever attending the menstrual flux. This being the case, it is evident that a diagnosis cannot be made by simple observation without a careful study of all the symptoms. Again, in many women the causes for this condition are complex. It will not do to lose sight of this, and conclude that because a flexion exists, dilatation will remedy menstrual pain. It is to be remembered that if there is coëxisting pelvic inflammation, dilatation will increase it, and, under certain conditions, cause it if absent. Rapid dilatation of the cervix is a distinct traumatism, and along with it run all the dangers incident to septic absorption that attend any other violent procedure, and where traumatism, incident to natural causes, is confessed to be the cause of so much subsequent mischief, it ought not to be *expected that operative injury can be harmless.* This conclusion, reached inferentially, has been abundantly confirmed practically on the operating table by much of my later pelvic work. In a number of cases, with a history of preceding dilatation, the after-operation has exhibited an inflammatory condition of affairs as complicated as any other in my experience. Some of the dilatations were done with preëxisting disease, which was made worse by this interference, while others were done simply to relieve the dysmenorrhea, and resulted in the establishment of a complicated surgical disease in which operation was *necessary purely to save life.* All in all, I believe that, judged simply by its remoter effects, the operation of rapid dilatation is a dangerous one, *and results oftener in subsequent harm than in lasting good.* The surgical injury to the cervix is, in many of these cases, more pronounced than the tears of the cervix which it is the intention to remedy by Emmet's operation. In this case there is operation at each horn of the dilemma, and the results are often equally bad at both. Simple closure of the cervix in cases of pelvic disorder almost certainly exacerbates the symptoms. The necessary inflammatory action set up in the suture tract is transferred along the lymphatic or venous channels to the seat of the earlier inflammation; this is lighted up anew and goes on in its development until a pelvic peritonitis is kindled or rekindled, which at last entails a major operation. The minor gynecologist, as such, who has no regard for, or appreciation of, the relation of the commonly advocated general closure of perineal and cervical tears to major surgical complications, cannot but be a great factor in the causation of the same. In Pepper's *System of Medicine*, vol. iv., there is on record a case in which the operator hoped to cure a pelvic inflammation by the derivative effect of a perineal or cervical operation. Needless to say, pelvic operation was afterward done. Such a cure is no less ridiculous than the so-called "faith cure," and is certainly more actively harmful.

That the inconsiderate use of the uterine sound has been responsible for much inflammatory pelvic trouble, is scarcely to be disputed. This is not because the sound is of itself a dangerous instrument, but because it is put into the hands of every tyro as an instrument of diagnosis. If used at all, it should be in the hands of those with whom its application, by reason of their skill, will be exceptional, not usual, and the rule should be that in the hands of the non-expert it should be forbidden. The more expert and experienced the specialist, the more rarely will the instrument be required. My own rule is, that in cases in which it might at first seem indicated, a little patience and diligence will obviate the necessity of employing it. The indiscriminate use of the sound and electrode is the most serious MECHANICAL objection to the employment of electricity. Every sitting for the electrical treatment is prefaced by the use of the sound, and followed necessarily by the introduction of an electrode of some form. This is by a class of men who, in the main, have had no *previous gynecological training or education* whatever. In such hands such methods can only be harmful, and we are now reaping the fruits of their work in a class of pelvic operations not surpassed in the complications presented.

Along with the sound and in the same category may be placed the curette. Dilatation, with curetting of the uterus, have placed to their credit a long *series of major operations.*

Another class of cases coming under this head are those in which there has been a long time during which intra-uterine applications have been made. All the caustics in the catalogue have at one time or another been in favor, as cure-alls, in intra-uterine therapeutics — nitric acid, chromic acid, nitrate of silver, and the rest. For a woman to have undergone a routine treatment with this list, and to have escaped pelvic inflammatory trouble, is little short of a miracle. A careful inquiry into many of the cases coming under my care, directly and indirectly, reveals the history that all sorts of minor procedures were tried, only to fail and apparently hasten the necessity for operation. I shall refer to and illustrate these points by the citation of cases in the discussion.

### DISCUSSION.

Dr. E. E. Montgomery: I fully second what Dr. Price has said with regard to the frequency of troubles necessitating major operations which result from the various methods of procedure in minor gynecology. I do not think that any person who has practised gynecology has not met with cases of inflammatory trouble of the uterus traveling to the ovaries and to the peritoneum, giving rise to conditions which have been described as peri- and parametritis, which have resulted from the use of the uterine sound. When we consider the fact that the uterine sound has been a part of the routine method of examination of many physicians practising this branch of the profession, it is not surprising that these troubles should so frequently occur. The uterine sound, as has been stated, should not be introduced in any case until the patient has been thoroughly examined, and the presence or absence of any inflammatory condition in the uterus, or about it, has been eliminated.

The practice of Emmet's operation upon cases as soon as they consult a physician for treatment, where a slight laceration is found and the physician at once attributes the symptoms to this lesion and performs the operation, has justly led to its discredit. The operation is undoubtedly one which, in some cases, is of great benefit; it is so, however, in properly selected cases. No case in which the presence of other inflammatory conditions has not been eliminated or cured by proper methods is suitable for the operation. One reason, I think, why Emmet's operation has proved so disastrous in many of these cases is the fact that, as the result of sub-involution of the mucous membrane from this lesion, we have an increased amount of secretion which, after narrowing of the cervical canal by the operation, is unable to escape freely ; consequently, the uterus becomes dilated to a certain extent, and this favors more rapid extension into the Fallopian tubes, and the development of serious trouble. One cause of the extension of inflammatory trouble from the uterus to surrounding parts is insufficient drainage from the cavity of this organ.

Then, again, Dr. Price very justly condemns the use of irritating materials, which have been employed in the cavity of the uterus. Many who have proposed agents for the treatment of inflammatory troubles in the cavity of the uterus have seemed to labor under the idea that the only method of curing these inflammatory lesions was by destroying the mucous membrane in which they originated. The application of nitric acid, chromic acid, nitrate of silver in stick, and the like, results in relief by destroying the mucous membrane from which the secretions take place. In this way inflammation may be caused which may extend to the deeper structures of the uterus and to the pelvis. I fully agree with all that the gentleman has said in regard to the importance of care in the treatment of these various classes of cases, to avoid adding to the discomfort and to the crippling of the whole future of the individual who applies for treatment.

Dr. John C. DaCosta: I am glad to hear Dr. Price speak of the dangers of minor gynecology, but I do not know how we shall get along without it, unless we adopt the rule (which he leads us to infer from his paper is his) that in all these ailments we open the abdomen and remove the tubes and ovaries. I hardly think that Dr. Price is right in attributing the major pelvic troubles to gynecological treatment, for, from the little that he has said, I think that we may infer that the pelvic trouble already existed, and the practitioner made a mistake in treating the uterus rather than the uterine appendages. I am glad to hear him speak in regard to Emmet's operation. I have heard long lists of cases reported with the statement that "all recovered without bad symptoms." That has not been my fate. One of the hardest fights that I have had for a woman's life has been after an operation on the cervix. Many unnecessary operations are done on the cervix. They are often done by men who want to make a record — by men who practise gynecology without knowing much about it. They see a torn cervix, and, without knowing whether or not the symptoms are due to that, proceed to operate. They do it, also, without properly preparing the patient beforehand. Where a lacerated cervix needs operation, as a rule, it needs previous treatment. If the cervix is put in proper condition, there will not be the same liability to bad results.

There is probably no instrument that is more used in minor gynecology than the dilator, and there is probably no instrument that can be more abused than the dilator. Prof. Goodell has reported to this Society many cases in which forcible dilatation has been used with grand results. I have used forcible dilatation in many cases and have never had any bad results. The reason is that when I began the study of gynecology, I was taught how to use it properly and not to use it in every case. Take the sharply bent womb, and all the pessaries made will not straighten it. You must put something inside, either a dilator or a sponge-tent. Again, let the uterus become congested and the mucous membrane swollen, closing the uterine canal and causing dysmenorrhea. You can cure that case in from two to four treatments by dilatation, while you may treat it by other means for months without doing good. The dilator is a surgical instrument, and one which must be handled carefully. You must know how to do your work before you attempt to use it.

Now, in regard to the use of the sound. I hear gentlemen state that they can outline any uterus without the sound. I have tried that, but have never been able to do it. Take a uterus enlarged, like this sketch, and I defy any one to say in what direction the canal runs. It may be a uterus in the normal position, with a fibroid in the posterior wall, or it may be a retroflexed uterus with a fibroid on the anterior wall, or a plastic mass between the uterus and bladder. It behooves us to use the sound carefully. If a man tries to force the sound into the canal, he will certainly do damage. If, however, he will outline the shape of the uterus as well as possible, and then bend the sound to fit as nearly as may be, and then make effort after effort, he can, in the most distorted uterus, get the sound in without damage.

Then, in regard to the curette. These usually have a sharp cutting edge. Such an instrument is hardly safe for an able practitioner to use, and is not safe at all in the hands of an unskilled person. Where inflammation extends from the uterine cavity to the tubes, after the use of the curette, it is not so much from the instrument as from the man who uses it.

I should be loath to give up intra-uterine applications. I have used them a long time, and, while sometimes pain has been caused, they have never done any serious damage. As Professor Wallace used to say, "Some uteri are sensitive to the slightest touch, and some are as stupid as oxen." When you make an application you must know the uterus which you are treating. Nitrate of silver used to be a common application, but it is one of the worst that you can make. It will, as a rule, produce cicatricial contraction of the canal. Nitric acid, although so much stronger than nitrate of silver, is not so apt to do this ; but nitric acid is rarely required. In a case of fungous granulation I should not hesitate to scrape out the whole inside of that uterus and make a strong application, and, after watching the patient for a short time, send her home and not expect to have any trouble. This is because I know my cases. I do not do it in every case.

I think that Dr. Price will find that the dangers from minor gynecological operations are more because of want of good, sound judgment in the practitioner, and not so much in the operation itself. I cannot agree that pelvic troubles are always due to these minor operations.

Dr. Joseph Hoffman: I have put on record in the Obstetrical Society a case where the uterus was perforated by the curette, and this case serves to show that the remarks of Dr. DaCosta enforced the argument which Dr. Price endeavors to bring out, to-wit, the danger from the wide-spread use of the uterine sound, the curette, and the dilator, as advocated by some. I believe that if we took all the gynecological instruments invented and put them together and multiplied them by ten, we should have no such instrument as gives such bad results as the dilator. It is easy for Dr. DaCosta to claim that he knows when to use it and when not to use it. I think that he over-estimates his ability to say whether he has ever done harm by it, for patients rarely come back after they are harmed. I have seen today two patients that had been treated by the curette, and from whom I have removed the appendages. In one case that I know of, the uterus was torn by the dilator, then a sponge-tent was put in and allowed to remain I do not know how long. You know the rest. In the case in which the uterus was perforated by the curette, the operation was done by a gynecologist of considerable experience. Nevertheless, the uterus was ruptured and peritonitis was brought on and abdominal section was necessary to save life. I have today seen two other women who were treated by minor gynecology; they were both left very miserable. In one the vagina is much contracted and the pelvic viscera are certainly affected. In one of these cases, especially, electricity was used *ad nauseam*. The history is this: first, dilation and scraping; then, closure of the perineum; and then, opening of the abdomen. In regard to operations on the cervix and perineum, we are to remember that operation on the perineum is not so apt to cause trouble as operation on the cervix.

What operations on the cervix are necessary? Every cervix with a slight laceration does not require operation. Some of these heal without suture, although traces of the damage may remain. The preparation of the patient often shows that operation is unnecessary — puncture and the ordinary derivative procedures so reducing the size of the cervix that the laceration almost disappears.

In regard to ulceration of the cervix, I do not believe that there is such a thing, except as the result of bad laceration or specific disease. In laceration the ulceration is only apparent; it is really an erosion due to eversion and hypertrophy.

The curette in some cases seems to be a necessary evil which we cannot do without. I have found it useful in getting rid of putrid *débris* from a miscarrying uterus, in the early weeks of pregnancy, when the use of the finger is thoroughly clumsy and painful, if not impossible, without previous dilatation with a tent. In the presence of such detention, the use of the tent is not without danger, since, during the period of its presence in the cervical canal, the channel of escape for decomposing material is shut off. I can say that I have had no bad results, that I know of, in the use of the instrument.

As to the use of the sound, its use seems more confined to those who are wedded to the traditions of the instrument than from any *actual* value that can be attached to it. On the other hand, it is capable of doing much harm in the hands of those who need it most, because they know least about it and the parts with which it has to deal.

Dr. William E. Ashton : The question of the use of the dilator depends upon one or two facts. First, as to the condition of the uterine appendages and their surroundings ; and, secondly, properly selected cases. I do not imagine that anyone would use the dilator when we have present acute or chronic inflammation of the uterine appendages. I think that anyone who has had experience in the use of the uterine dilator would hesitate to employ it except in selected cases. I believe that where we have the pelvis perfectly free from local disease, and in cases where the uterus is strongly anteflexed and perfectly movable, and upon the introduction of the sound we find that there is a point of intense pain at the internal os, we shall find in a certain proportion of cases that good results are obtained from the dilator. It is nonsense to talk about the causes of dysmenorrhea. It is only a symptom. The vast majority of cases of dysmenorrhea are cases which have a distinct tubal or ovarian origin. It would be absurd to rapidly dilate in such cases.

In regard to Emmet's operation, I quite agree with Dr. Price in reference to minor gynecological operations dealing most disastrous results in the pelvis. We have to look only at the various clinics, and see the recklessness with which various operations are done, to see why we have so many abdominal sections. The reckless use of the sound and of uterine applications are responsible for many of these cases. I hold that the sound should only be used after a diagnosis is made. If the diagnosis is made, of what use, then, is the sound ? In a case like that figured by Dr. DaCosta, I do not care what direction the uterine canal takes. If it is a fibroid, I do not see of what use such knowledge would be.

I cannot understand how any man can use instruments where there are inflammatory conditions around the pelvis, because they are a source of irritation and may light up acute inflammation in chronic cases. It should go on record that the uterine sound should only be used by men who have a thorough knowledge of the pathology of the pelvis, and who can appreciate the great danger incident to inflammatory troubles in the pelvis.

I agree with Dr. Price that there are few cases in which Emmet's operation is necessary. I grant that there are cases in which the uterus is in a state of subinvolution, where a plastic operation will bring about the cure, but I do not believe in operating on the uterus if there is any diseased condition of the appendages. Any manipulation under such circumstances is apt to set up inflammation. Four years ago, I had a case of bad laceration of the cervix in a woman, with pus tubes on both sides. I refused to operate on account of the disease of the appendages. She then went to New York, and was operated on by a prominent gynecologist, and died of large abscess, the result of the operation lighting up the old inflammation.

Dr. DaCosta, in answer to Dr. Ashton : I probably did not make myself clearly understood. I do not want it understood that I would do an operation on the cervix if there was inflammation in the pelvis, such as a pus tube or anything else. My teaching is : When there is violent inflammation in the pelvis, not to do any operation on the uterus, and to hesitate to use the sound. The discussion has run off from the original text, and it is for that reason that I make these additional remarks.

Dr. J. M. Baldy : I think that there is no question in the minds of gynecologists that Emmet's operation is a much abused operation. I think that it is also true that the vast majority of these ill-advised operations on the cervix are done by men who have no gynecological experience and who know very little about gynecology. I can recall two cases in which I was recently called to operate on the cervix by general practitioners, and by whom I was informed that the lacerations were very bad and the women were suffering greatly. On examination, the tears proved to be comparatively slight, and needed no interference. There are some cases in which a cervix operation at first sight appears justifiable. These are cases in which the cervix is torn to the vaginal vault. I care not if the cervix be torn on both sides to the vaginal vault, if there is not eversion and erosion, or much scar-tissue, there is no reason for operation.

I should be loath to give up forcible dilatation in certain cases. It is not to be done in every case of dysmenorrhea, for the vast majority of cases of dysmenorrhea are due to ovarian or tubal disease. I believe that in the vast majority of cases where trouble follows the use of the dilator, there has been preëxisting pelvic trouble. I do not think that a carefully done dilatation in a healthy pelvis will do harm. It is admitted that it does tear uterine tissue, but that this can cause trouble, unless the wound becomes septic, I am not prepared to admit.

The use of the sound in the hands of a doctor is in inverse proportion to his skill. The man who is skilled, rarely uses it. In such cases as have been mentioned by Dr. DaCosta, I see no use for the sound. I do not see anything essential that it could tell. I must say that I have not seen a uterus of the exact shape he has figured on the board. In the vast majority of cases I have been able to tell which was fundus and which was tumor. If we are dealing with a fibroid, it makes no difference what wall of the uterus it occupies. I do not suppose that I use the uterine sound once a month.

The curette, I think, is a valuable instrument, but it is abused and used indiscriminately. After abortion, I find it most valuable. In some cases of chronic endometrial disease it is valuable. I believe that it will remove almost all necessity for intra-uterine treatment. I find such applications rarely called for, except, perhaps, the application of nitric acid or iodine, after the use of the curette. I think that the dull curette is useless. The only rational instrument to use is the sharp curette. I was recently called some seventy miles to see a case where the physician assured me that the uterus contained nothing, as he had twice gone over it thoroughly with the dull curette. I used a sharp curette and removed large masses of placental tissue. The sharp curette can be used with as little danger as any other instrument, if used properly in skilled hands.

Dr. J. M. Fisher: I am engaged in treating a number of uterine troubles with electricity. Dr. Price has stated that the use of the electrode is fraught with much danger. That the introduction of any instrument into the uterine cavity carries with it a certain risk is not denied, but that the electrode is especially responsible for many of the diseases can certainly be questioned. There are certain diseases of the uterine tissue and lining membrane that can be most effectively treated by properly applied galvanism. I can cite one case in which the use of electricity saved the patient from undergoing a major pelvic operation. A woman, forty-two years of age, had a fibroid uterus with hemorrhages, so that she was confined to bed half the days of the year. At the time that I was called she had been laid up for nine weeks and was exsanguine from loss of blood. She had been treated by two good practitioners, and, failing to give her relief, operation was proposed and about to be done. I made a positive application of electricity, and, after the first or second application, the hemorrhage was arrested. Four applications, extending over a period of twenty-one days, were made. This was in November and December, 1889. After that she menstruated regularly until May, when she was again seized with hemorrhage. I was out of town, and the bleeding continued three weeks. On my return, I made positive application of electricity, and since then the menstrual discharge has been regular, lasting three or four days.

Dr. C. P. Noble: I am glad that this matter of the uterine sound has been brought up, because I am convinced, as the result of my experience, that the less the uterine sound is used, the better for the patient. In most cases but little information is gained. Recently a case passed through my hands in which the question of pregnancy was mooted. She afterwards fell into other hands, and the sound was passed three inches and the patient was supposed not to be pregnant. She was, however, seven months pregnant, as subsequent events showed. The information given by the sound is often delusive. I, however, cannot see that the simple passage of the sound, provided it be clean and passed through a speculum, with a clean cervix, should set up pelvic inflammation, provided such trouble does not already exist. This, however, is neither here nor there, for I do not see that we need to use the sound in diagnosis. In small uteri it is not needed, because the organ can be outlined bimanually ; while in large uteri, where tumors are present, the instrument may not reach the fundus and so give incorrect information.

I must agree with Dr. Baldy, rather than with the author, in regard to rapid dilatation. I should be loath to give it up. I have never seen harm follow rapid dilatation in any case. This is due to the fact that dilatation has been used in cases in which the disease is limited to the uterus. I agree that it is useless and dangerous to dilate the uterus when tubal disease is present. In uterine disease it is capable of doing a great deal of good. I am quite sure that a certain number of cases of tubal disease are set up by a narrow cervix. The secretions of the uterus cannot gain egress and set up endometritis, and the inflammation travels into the tubes. In these cases, if the cervix is dilated to allow the freer egress of secretions, it will be a positive factor in the prevention of tubal inflammation. In such cases as were mentioned by Dr. Ashton, of acute anteflexion, the dilator does a great deal of good.

In fact, in regard to all these minor measures which have been mentioned tonight, I find them of service, but the fact must be emphasized that they are useful only when the disease is limited to the uterus ; and that the uterus should not be operated on, in any way, in the presence of pelvic inflammation, particularly abscess.

Why we should give up the curette I cannot understand. There are many cases of hemorrhage from the uterus due to uterine disease purely, where there is no ovarian or tubal disease. In such cases the use of the curette will permanently control the hemorrhage.

I think that one reason septic troubles follow minor operations is because antiseptic precautions are not observed. I think that is the case with the dilator. If used on the office table, it is impossible to employ complete antisepsis. If such precautions are used, and there is no extrauterine inflammation present, I do not think that inflammation will follow any of the minor gynecological operations.

Dr. Price : I am sorry the discussion has taken the direction that it has, for it does not give me an opportunity to express myself thoroughly ; it does not give me an opportunity of pulling out a number of telegrams ; it does not give me an opportunity of calling things by their right names. I have thrown down the gauntlet, and no one has quite taken it up. Some one has spoken of minor gynecological methods.

In a recent article a writer prefaces what he has to say by giving details of methods for the treatment of "ordinary gynecological troubles." I do not know what "ordinary" gynecological troubles are. If it means from nine o'clock to three in an office, with a nurse and a Sims's speculum, peeping at cervices, and taking ten dollars from each patient, then I understand it. He is the great mischief-doer. He tinkers, dilates, curettes, and passes the sound, and in from four to six weeks I get a telegram to come and open the abdomen to save the patient's life; that the woman is leaking; that she has a pulse of 130-140, with a temperature of 104 degrees. This occurs weekly,

A speaker stated that there is no harm in electricity. Three fibroids in that jar have pus in them as the result of the use of electricity. Of the twenty specimens in that jar, removed during August, fifty per cent. followed dilatation, closure of the cervix, the use of the sound and the curette. These specimens have come from four clinics in this city, and from ten prominent gynecologists. They all had sections done to save life, and all were greatly complicated operations.

In regard to the sound, Dr. Ashton has said all that I am capable of saying. I have not used the instrument for many years. It is a common method of determining the existence of pregnancy, particularly among homeopaths, although not confined to them.

In comparison with the former state of the same subject, we must inquire into the causes which must have been at work during the past few years. This private-office work has a great deal to do with it. Many of these men are simply cervix feelers, and never find anything above it. There may be a mass larger than the uterus on one or both sides, which they fail to find. They are not anxious to find them, and would not be troubled by them.

The dysmenorrhea in an infantile uterus has nothing to do with the uterus. Pelvic pain in all infantile conditions of the uterus and pelvic viscera is exceedingly common. In these cases dilatation avails nothing. Dr. Baldy says that he uses the sound once a month. I presume that he dilates about once a month. I will consider together drainage of the uterus, referred to by Dr. Noble, and the use of the sound and dilator, referred to by Drs. Baldy and Noble. The sound measures about two lines in diameter, but we will say that it measures only one, I am sure that the drainage is quite sufficient through a canal one line or more in diameter. I find that those who have such a love for dilatation always precede it by the use of the sound. If they use it for drainage, the indications are not clear.

Dr. Baldy: I would ask to what part of my remarks Dr. Price refers. He has entirely misunderstood me. He stated that presumably I dilated for drainage, and that I first pass the sound, which will of itself establish drainage, without the dilator. My remarks were not in regard to dilatation for drainage or anything of the kind. I do not know that I specified what I would dilate for. Time did not admit of my discussing that point. In regard to passing the sound once a month, I do not know that I meant to make that a positive statement. The statement was simply made to illustrate the infrequency with which I use the sound.

Dr. Price : I thought that I had made that clear. I said that I would call attention to two points — that of drainage, as referred to by Dr. Noble, and the sound and dilator, as referred to by Dr. Baldy.

In regard to closure of the cervix, there are a few cases in which the operation is of importance, but the ordinary method of closing the vaginal surface of the cervix only is very imperfect. This forms a large cuspidor-like cavity or retention sac. I have repeatedly split these up, freshened the cervix, and made a perfect cure.

I have thrice this Summer been called out of the city to open the abdomen in cases in which dilatation had been performed a short time previous.

Disease of the cavity of the uterus and fungous vegetations are far from common. Many healthy uteri are curetted, and it is thought that granulations are found. If the woman had been let alone she probably would have conceived. The same is illustrated by a class of cases which I have studied among women locked up in a reformatory. Some twenty or thirty women, who had been living lives of chronic inebriety and lust for three or more years, had none of them conceived. After six months' rest, iron and good diet, the greater number conceived on leaving the institution. In these cases no intra-uterine treatment was employed, and only one examination was made to determine the position of the uterus and its relation to surrounding parts in the pelvis.

As a diagnostic instrument, I do not see why any one should want to use the sound. As a student, I never could see what was gained by the use of the sound ; in the hands of the trained or experienced it is not needed, and in the hands of the inexperienced it is dangerous. Too much prominence is placed upon an unhealthy condition of the uterus ; it does not often exist ; it is exceptional.

